# Caffeine supplementation improves the cognitive abilities and shooting performance of elite e-sports players: a crossover trial

**DOI:** 10.1038/s41598-024-52599-y

**Published:** 2024-01-24

**Authors:** Shih-Hao Wu, Yu-Chun Chen, Che-Hsiu Chen, Hou-Shao Liu, Zhi-Xin Liu, Chih-Hui Chiu

**Affiliations:** 1 School of Medicine, College of Medicine, China Medical University, Taichung, Taiwan; 2 Department of Emergency Medicine, China Medical University Hospital, Taichung, Taiwan; 3 Department of Physical Education, National Taiwan University of Sport, Taichung, 404 Taiwan; 4 Department of Sport Performance, National Taiwan University of Sport, Taichung, Taiwan; 5 Graduate Program in Department of Exercise Health Science, National Taiwan University of Sport, No.16, Sec. 1, Shuang-Shih Rd., Taichung, 404 Taiwan; 6 Institute of Sports Sciences, University of Taipei, Taipei, Taiwan

**Keywords:** Neuroscience, Physiology

## Abstract

We explored the effect of 3 mg/kg of caffeine supplementation on the cognitive ability and shooting performance of elite e-sports players. Nine e-sports players who had received professional training in e-sports and had won at least eighth place in national-level e-sports shooting competitions. After performing three to five familiarization tests, we employed a single blind, randomized crossover design to divide participants into caffeine trial (CAF) and placebo trial (PL). The CAF trial took capsules with 3 mg/kg of caffeine, whereas the PL trial took a placebo capsule. After a one-hour rest, the Stroop task, the visual search ability test, and the shooting ability test were conducted. The CAF trial’s performance in the Stroop task in terms of congruent condition (*P* = 0.023) and visual search reaction time with 20 items (*P* = 0.004) was significantly superior to those of the PL trial. In the shooting test, the CAF trial’s kill ratio (*P* = 0.020) and hit accuracy (*P* = 0.008) were significantly higher, and the average time to target (*P* = 0.001) was significantly shorter than those of the PL trial. Caffeine supplementation significantly improves e-sports players’ reaction times and shooting performance.

## Introduction

Owing to the popularity of e-sports, numerous countries have recognized e-sports as a formal sport. E-sports were also included as a demonstration event at the 2018 Asian Games in Jakarta and as an official event at the Hangzhou Asian Games in 2023. Professional e-sports leagues have been founded in several countries. E-sports include multiplayer online battlefield arena games (e.g., *League of Legends*), first-person shooter games (e.g., *Counter-Strike: Global Offensive*, and *Valorant*), real-time strategy games, and sports games^1^. For example, Game for Peace is a first-person shooting game and an official sport of the 2023 Hangzhou Asian Games. Studies on first-person shooters have revealed that players with experience in competitions had shorter reaction times in a Stroop test^2^ and stronger visual search abilities^3^. Therefore, improving cognitive abilities that relate to e-sports competitions may be critical for participants in first-person shooter competitions, and external nutrients that enhance cognitive abilities may be crucial to their success.

Caffeine is a popular and effective ergogenic supplementation for athletes of all levels^4,5^. It is typically consumed through food and drink, and the mechanism through which low-dose caffeine acts as a psychostimulant is based on central antagonism at the A_1_ and A_2A_ adenosine receptors. The capacity of caffeine to bind adenosine receptors facilitates the inhibition of the brake that endogenous adenosine imposes on the ascending dopamine and arousal systems, which facilitates cholinergic and dopaminergic transmission^6,7^. Therefore, caffeine consumption may improve energy, mood, cognitive function, attention, simple reaction time, choice reaction time, and memory and alleviate fatigue^8,9^. The consumption of caffeine 1 h before playing a first-person shooter can improve players’ visual search ability and speed in a state of alertness^10^. A dosage of 3 mg/kg of caffeine before a game can increase players’ typing speed^11^, shorten their reaction times and increase their shooting accuracy^12^. However, few studies have explored the ergogenic effects of caffeine on problem-solving abilities by Stroop task^13^ on Esports players.

This is because visual search ability and the presence of decoys that distract the player's attention both affect performance in first-person shooting games. When investigating the effect of caffeine on shooting accuracy, it is not sufficient to use simple reaction time as an indicator of cognitive ability. Therefore, the purpose of this study was to explore the effect of caffeine intake 1 h before a first-person shooting game on players' performance by using the Stroop task and testing their visual search and shooting abilities.

## Methods

### Design

The study was a single blind, repeated-measure, crossover design where participants were randomized to ingest a caffeine capsule (CAF) and a placebo capsule (PL) separated by 7 days, 1 h before performing cognitive function tests. We used computerized randomization to arrange the order of participants in the experiment. At least 1 week before the formal experiment, all subjects participated in three to five familiarization tests, such as cognitive function and shooting tests. The primary outcome was the results of the cognitive function tests, and the secondary outcome was shooting performance. The study started on 01/01/2022 and ended on 30/04/2022.

### Participants

We recruited nine healthy male adults (age: 20.8 ± 0.9 years; height: 172.3 ± 1.2 cm; mass: 72.8 ± 8.3 kg; training age: 2.8 ± 0.3 years). All participants have experienced international first-person shooting and are recruited from national Esports training centers. We did not recruit female participants to eliminate the effect of their menstrual cycle, which could have increased the confounding factors of the experiment^14,15^. The inclusion criteria were: (i) healthy male adults, those individuals who are free of pain, insomnia, or other injuries recently, without any medication used in recent 2 months, (ii) underwent training (more than 5 days per week) in first-person shooters, are a type of shooter game^16^ that relies on a first-person point of view with which the player experiences the action through the eyes of the character, more than two years. The exclusion criteria were: (i) females, (ii) below 20 years old, (iii) did not have sufficient training/competitions experience (for example, did not have experienced international first-person shooting), (iv) with cardiovascular diseases or any disease that made subjects feel ill, (v) participants with daily caffeine intake below 80 mg^12^. According to the pre-test dietary recorded by photos, the participants had an average daily caffeine intake of 44.1 ± 32.9 mg. Among the participants, the lowest mean daily caffeine intake was 0 mg and the highest was 78 mg.

Two weeks before the main trial, all the participants were asked to avoid ingestion of more than 80 mg of caffeine a day. Before the experiment, all participants were fully informed of the experimental procedures and risks and provided informed consent. All the study was executed in the eSports room. This study received approval from the Institutional Review Board of Jen-Ai Hospital-Dali Branch (111-06) and registered in the ClinicalTrials.gov (Date: 30/08/2022; ID “NCT05521347”; https://register.clinicaltrials.gov). This study was conducted following the Declaration of Helsinki.

### Protocol

#### Experimental procedure

All tests were conducted in a professional e-sports classroom, and the indoor ambient temperature was set at 26 °C. All participants' computers and chairs were equipped with the equipment they were most accustomed to training on. Participants’ diet and mealtimes were recorded for the 3 days before the first formal experiment, and the participants were required to follow the same diet 3 days before the next formal experiment. Nutrient composition on Day 1 included 11.7 ± 4.1% protein, 42.6 ± 13.3% carbohydrate, 28.9 ± 6.8% lipid and 2265.0 ± 265.6 kcal. Nutrient composition on Day 2 included 11.4 ± 2.3% protein, 40.0 ± 10.6% carbohydrate, 28.9 ± 6.1% lipid and 2050.0 ± 454.4 kcal. They were also required to avoid food and beverages with caffeine (e.g., coffee, energy drinks, chocolate, chocolate drink, and tea) 3 days before the formal experiment.

On the day of the formal experiment, participants had breakfast and lunch at 8:00 a.m. and 12:00 p.m., respectively. The nutritional composition of breakfast and lunch was 10.9 ± 3.5% protein, 42.9 ± 15.9% carbohydrate, 26.3 ± 7.8% lipid, and 1190.2 ± 235.4 kcal. The participants arrived at the classroom at approximately 3:00 p.m. for the experiment. The participants took capsules with 3 mg/kg of caffeine (CAF trial) (caffeine, Wako Pure Chemical Industries, Ltd., Osaka, Japan) and or a placebo capsule (PL trial) with 200 mL of water. The placebo capsule contained flour. All the capsules used for the placebo and caffeine trials are identical in size, shape, color, and taste. Each participant had a computer with a frame rate of at least 240 Hz and a mouse with a scrolling speed of 1 ms. After the participants remained in the room for 1 h, during which time they were asked to take any form of rest other than engaging in e-sports, they took the Stroop task, the visual search test, and the shooting ability test. All participants completed all tests without adverse effects.

#### Outcome measure

The color-word Stroop task and visual search test were conducted using Psych/Lab for Windows. Measures used in the literature have satisfactory reliability and validity^17^. The Stroop task involved four base colors, namely red, green, blue, and yellow, and the names of the base colors were presented in Chinese characters and in diverse colors to confuse the participants. The participants were required to press a key corresponding to the base color name they saw on the screen (“R” for red, “G” for green, “B” for blue, and “Y” for yellow). In each test, eighty trials lasting 5 min each were conducted. The test results comprised a congruent condition, in which the key pressed corresponded correctly to the color on screen, and an incongruent condition, in which the key pressed corresponded incorrectly to the color name on screen.

In the visual search test, participants identified orange “T” s on the screen from upside-down orange “T” s, blue “T” s, and upside-down blue “T” s. When an orange “T” would appear, the participants were required to press the spacebar as quickly as possible. If no orange “T” appeared, the participants were required to not react. A total of 80 search displays were presented in 5 min. In each display, 5, 10, 15, or 20 items were presented.

The shooting ability test involved a three-dimensional aim trainer, which was proposed in a previous study^12^. Participants used the mouse to shoot the electronic targets on the computer screen. The test comprised sixty targets and could be completed in 2 min. It was performed on a static map with medium difficulty. In each round, three targets appeared at once, and participants were required to shoot them within 2 s, ideally eliminating each target with a one-shot kill. Participants’ kill ratio (number of targets hit/60), hit accuracy (60/number of shooting), and average time to target were noted.

### Statistical analysis

All data are presented as averages ± standard deviations. The Shapiro–Wilk test was used to examine the normality of the data. Cognitive performance, accuracy, and hit reaction time were analyzed through a paired sample t test. We used G*power 3 software 24 to achieve an alpha value of 5% and a power of 0.8; a sample of six was considered sufficient for this study. Effect sizes were calculated using Cohen's D. All data were calculated using SPSS (version 20, Chicago, IL, USA), and the significance level was α < 0.05.

## Result

### Stroop task

**Figure 1:**
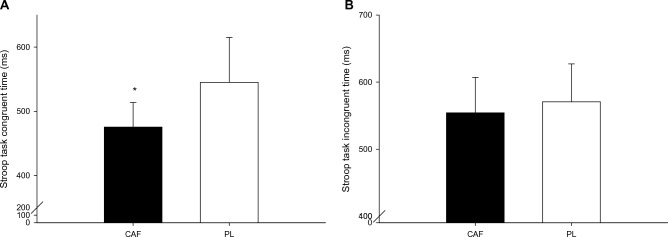
The reaction time in the congruent condition of the Stroop task (**A**) and the reaction time in the incongruent condition of the Stroop task (**B**). *CAF was significantly higher than those for the PLA (*P* = 0.023) in the congruent condition of the Stroop task.

The reaction time in the congruent condition of the Stroop task (Fig. 1A) of the caffeine trial was significantly shorter than that in the placebo trial (*P* = 0.023). The reaction time in the incongruent condition of the Stroop task (Fig. 1B) did not significantly differ between the trials (*P* = 0.478). The effect size (Cohen’s D) was 1.2 for the congruent condition. The correct rates were not significantly different in congruent condition (CAF: 89.2 ± 18.6%; PLA: 87.3 ± 9.2%; *P* = 0.715) and incongruent condition (CAF: 93.8 ± 5.7%; PLA: 86.7 ± 5.3%; *P* = 0.273).

### Visual search reaction time

The visual search reaction time (Table 1) in the caffeine trial was significantly shorter than that in the placebo trial (*P* = 0.020) with 20 items. The effect size (Cohen’s D) was 0.95. The visual search reaction time did not differ significantly between the trials (*P* > 0.05) with 5, 10, and 15 items.

**Table 1:** Visual search reaction time (ms) for CAF and PL.

	CAF	PL	*P* value
5 items	520.6 ± 49.9	505.0 ± 45.8	0.309
10 items	601.0 ± 63.2	614.0 ± 110.0	0.778
15 items	652.1 ± 79.3	618.9 ± 77.8	0.410
20 items	637.0 ± 101.1	728.0 ± 91.9	0.004*

Values are mean SD, n = 9. CAF, caffeine trial; PL, placebo trial.

*CAF was significantly lower than those for the PL.

### Shooting performance

**Figure 2:**
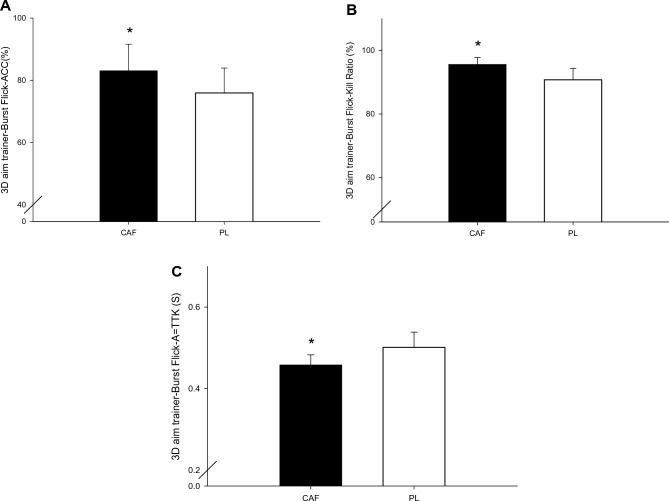
The kill ratio (**A**), kill accuracy (**B**), and the average time to target (**C**) of the shooting ability test. *CAF was significantly higher than those for the PLA in the kill ratio (*P* = 0.020), kill accuracy (*P* = 0.008), and the average time to target (*P* = 0.001).

In the caffeine trial, the kill ratio (*P* = 0.020) and accuracy (*P* = 0.008) were significantly higher than those in the placebo trial, and average time to target (*P* = 0.001) was significantly shorter (Fig. 2). The effect size (Cohen’s D) was 0.96 for kill ratio, 1.6 for accuracy, and 1.96 for average time to target.

## Discussion

The aim of this study was to investigate the effect of supplementary caffeine with 3 mg/kg on improving performance of E-sport players in Stroop task, visual search reaction time, kill ratio, hit accuracy, and average time to target. Caffeine supplementation improved reaction times in the congruent condition of the Stroop task, visual search reaction time with twenty items, kill ratio, accuracy, and average time to target, but the effects on reaction time in the incongruent condition of the Stroop task were insignificant.

Caffeine exhibits dose-dependent effects with desirable effects at lower doses (i.e., ≤ 400 mg) and detrimental effects above this level, although there is considerable inter-individual variation. For example, at doses of 250 mg, increased arousal, alertness, concentration, and well-being have been noted in human subjects^18^. Concentrations of 3–6 mg/kg caffeine are considered safe^19^ and helpful for the performance of E-sport players^12^. In our study, caffeine supplementation at 3 mg/kg showed a clear benefit on the various measures described above. In contrast, increased tension, nervousness, anxiety, excitement, irritability, nausea, paresthesia, tremor, perspiration, palpitations, restlessness, and possibly dizziness occur at a dose of 500 mg^20^. These effects may interfere with E-sports athlete performance, especially under fast-paced, high-stress visual and auditory stimuli. Ebrahimi et al. had proved that taking 5 mg/kg of caffeine can increase the blood pressure and heart rate of the shooters that leads to a decrease in shooting performance^21^. Sub-lethal doses of 7–10 mg/kg produce symptoms such as nausea, headache, chills, flushing, palpitations, and tremor, although individuals’ responses may vary significantly^18^. In extremely high doses, especially for some vulnerable populations, caffeine consumption could be harmful, including seizure or impairments in cardiovascular function, such as hemodynamic collapse and refractory arrhythmia^22^, however, it is extremely rare^23^. In 2017, the American Association of Poison Control Centers reported 3765 cases of caffeine overdose, of which 650 were intentional and none resulted in death from caffeine alone. Ingestion of 5 g (80–100 mg/kg) is likely to prove fatal^24^. Therefore, caffeine with 3 mg/kg is a safe psychomotor-activating supplementation for general populations, and a popular and effective ergogenic supplementation among athletes.

To the best of our knowledge, only one study has explored the effect of caffeine supplementation on e-sports players’ cognitive and shooting abilities. That study revealed that caffeine can improve attention, reaction time, and shooting ability, which is consistent with our results^12^. However, that study only involved a reaction test, the results of which might not explain improvements in cognitive function. The results of the Stroop task and visual search test in our study explain why caffeine improves shooting ability. We discovered that caffeine increases cognitive speed and shortens reaction times. The test with the aim trainer also revealed significant differences, indicating that caffeine can improve shooting ability in e-sports. Our results suggest that caffeine supplementation affects not only reaction time but also complex visual search ability in first-person shooters.

In our study, caffeine supplementation with 3 mg/kg showed clear benefits in the reaction time of the Stroop task-congruent, however, insignificant effects in the reaction time of the Stroop task-incongruent in elite players of E-sports. Dixit et al. conducted a study on 30 male undergraduate students through tests before and 40 min after the students consuming 3 mg/kg caffeine. The caffeine did not affect performance in a Stroop task, but significant decreases in reaction time were observed^25^. Yuan et al. conducted a behavioral experiment on thirty-one healthy participants through a Stroop task before and after the participants drank approximately 210 mL of coffee. The response time in the incongruent stimulus condition was longer than that in the congruent and neutral stimulus conditions both before and after the participants consumed the coffee. A decrease in interference in the incongruent stimulus and an increase in facilitation in the congruent were evidenced by decreases in reaction time after caffeine consumption^26^. Hasenfratz and Battig observed short reaction times in a Stroop task after participants consumed 250 mg of caffeine^27^. These results are consistent with ours, indicating that caffeine supplementation can shorten reaction times.

In our study, caffeine supplementation with 3 mg/kg showed an obvious reduction in the visual search reaction time in twenty items, however, insignificant effects in the visual search reaction time in 5, 10, 15 times in elite players of E-sports. Lorist et al. administered 3 mg/kg of coffee to undergraduate students, which shortened response times, as in our study^28^. Significant effects were only noted in tasks with more items, which may be related to cognitive processing demands; a higher number of items would require more concentration. The caffeine facilitated concentration, and differences between the caffeine and placebo trials were only observed under certain cognitive processing demands. Durlach also revealed that 60 mg of caffeine from a cup of tea significantly shortened reaction times in a sample visual search^29^. Marsden and Leach revealed that caffeine can improve visual search abilities but not chart search abilities in experienced navigators^30^. Therefore, caffeine reduces visual search reaction times. Our results indicate that the effect of caffeine on reaction time is related to improvements in perceptual attentional processes rather than motor processes, as indicated by Saville et al.^31^. The results also suggest that caffeine is effective in improving performance in e-sports that require attention, accuracy and short reaction times.

This study revealed that caffeine can increase e-sports players’ accuracy in shooting moving targets and reduce their response times. This might be attributable to the 3-mg/kg dosage, which reduced reaction times in the congruent condition of the Stroop task and visual search tasks with more items. This might also be related to the ergogenic effects of caffeine on dynamic visual acuity and problem-solving abilities. Redondo et al. discovered that the effects of caffeine on visual search reaction times may be related to the ergogenic effect on dynamic visual acuity^32^. This is particularly relevant to our tasks, which require the accurate and rapid detection of moving targets. In first-person shooters, players use various strategies to react to quick processions of visual and auditory stimuli and adapt to changing environments^33^. Our tasks required the identification of stimuli and the appropriate responses, motor plans, and actions.

The metabolism of caffeine is highly variable among individuals due to different genotype expressions at the level of the CYP1A2 isoform of cytochrome P450, which metabolizes 95% of the caffeine ingested^34^. Southward et al. suggested that there might be up to 33% of those who do not enhance performance following caffeine ingestion (i.e., non-responders)^35^. Minaei et al. found that the ingestion of 6 mg/kg of caffeine improved peak power output only in participants with the AA genotype compared with the placebo trial; however, expression of the CYP1A2 did not influence average or minimum power output or fatigue index^36^. However, in our study, all our subjects could observe the effect of caffeine on those specific measurements.

This study used a rigorous, two-arm crossover, randomized, controlled design. All outcomes were measured using a computer to ensure reliability. Despite these strengths, this study has several limitations. The main limitation is the low external validity and that the AIM trainer needs to be assembled. However, the inclusion of participants with previous experience increased the external validity. To our knowledge, only one study used the aim trainer to evaluate the effect of caffeine on e-sports performance^12^. By using a computer-based aim trainer, this study ensured that the outcome measures accurately represented participants’ performance. The computers we used may have influenced the results. Each participant used dedicated computers, and we used rigorous criteria for frame rates and mouse speeds. Therefore, the results accurately represent changes in performance due to caffeine supplementation. In addition, there is considerable inter-individual variation in the effects of different doses of caffeine. Even at the same dose, some subjects may experience adverse effects such as palpitations, anxiety, or tremors, which can interfere with the performance of eSports, especially during fast-paced, high-stress visual and auditory stimuli. In our study, however, the dosage of caffeine was adjusted according to the subjects' body mass, and the method was accurate enough for a medical experiment. We did not measure caffeine levels during the experiment. However, we controlled the food diary for 3 days at both experimental visits and avoided food and drinks with caffeine during this period. Furthermore, there is a significant lack of female athletes in e-sports^37,38^. Although this makes it difficult for us to recruit female athletes, a woman's menstrual cycle may be one of the variables affecting experimental control^14,15^. For this reason, gender was specifically included as an exclusion criterion in this study. The fact that we only had male subjects did not affect the results of this study and its external validity.

In future studies, we may be able to differentiate between participants who consume caffeine and those who do not. In addition, we can supplement caffeine in different ways, such as chewing gum, sublingual tablets, etc., to see if these methods of consuming caffeine without drinking water are more beneficial to performance.

## Conclusion

Caffeine supplementation improved cognitive abilities, decreased reaction times in the congruent condition and visual search reaction times in tasks with more items, increased kill ratio and accuracy, and reduced average time to target among elite e-sport players. A dosage of 3 mg/kg of caffeine supplementation ingested 1 h before the game may have a positive effect on players’ performance in a first-person shooter by using a Stroop task and testing the players’ visual search and shooting abilities. Such findings may encourage coaches and athletes to consider the use of caffeine as a nutritional supplement prior to important competitions to further enhance competitive performance.

## Data Availability

All relevant materials are presented in the present manuscript.
